# Reduction of margins in external beam radiotherapy

**DOI:** 10.4103/0971-6203.41190

**Published:** 2008

**Authors:** Tomas Kron

**Affiliations:** Department of Physical Sciences, Peter MacCallum Cancer Centre, St Andrews Place, East Melbourne, VIC 3002, Australia, E-mail: Tomas.kron@petermac.org

The aim of radiotherapy is to deliver a very high dose of radiation to a tumor whilst trying to spare the surrounding normal tissues. Of course, it is impossible to spare tissues adjacent to a target completely — the very process of delivering radiation to the tumor will result in radiation passing through surrounding structures, and there will be scattered radiation resulting in dose to normal structures. However, it is also impossible to direct radiation perfectly well to a target, in the first place. Therefore, it is common practice to surround the clinical target volume (CTV) with a margin to allow for setup uncertainties and movement of the target. This results in the creation of a planning target volume (PTV), a process described by the International Commission on Radiation Units and Measurements (ICRU) in its reports 50 and 62.[1,2]

The ICRU considers two major components of the PTV margin:

- The internal margin allows for any variations in size and location of a target in relation to a reference point within the patient. Examples are motion of a lung tumor due to breathing or the volume changes of bladder cancer due to filling of bladder.
- The setup margin accounts for any uncertainty in aligning the internal reference point in the patient with the coordinate system (isocenter) of the radiotherapy treatment machine. Examples are daily variations in patient positioning, couch sag, and transfer errors from planning CT to treatment unit.

The two types of margins are typically considered to be independent of each other. Like other independent (or mathematically speaking, ‘orthogonal’) sources of uncertainty, they can be added in quadrature to yield an overall PTV margin. The consequence of this is that both components of the margin must be addressed in order to yield best results; reducing one component will only make the other more significant.

The aim of optimization in radiotherapy planning and treatment is to keep this margin as small as possible as it increases the volume of normal tissue irradiated. It is important to keep in mind that margins are to be applied in three dimensions—as such, even a small margin reduction can result in a significantly reduced irradiated volume.

Modern radiotherapy offers three major tools which may have an impact on the various uncertainties and therefore the PTV margins required.

1. Intensity-modulated radiation therapy (IMRT) provides the tool to deliver highly conformal radiation dose distributions.[3] In particular, for irregularly shaped targets that wrap around critical structures, IMRT allows conforming the dose to concave and convex surfaces alike. The excellent conformance is achieved by a highly complex delivery pattern, where each radiation field is subdivided into many segments.
2. Image-guided radiation therapy (IGRT) utilizes imaging in the treatment room prior or even during treatment to ensure that the target is in the correct spot.[4] Image guidance can have many different forms: the imaging tools available range from ultrasound to electronic portal imaging, planar x-rays, and computed tomography. As CT scanning is the most comprehensive assessment of patient anatomy, there have been several variations to the theme, with different manufacturers using megavoltage or kilovoltage x-rays to construct fan beam or cone beam CT image sets of the patient directly prior to treatment. The image quality of IGRT CT systems is typically inferior to the treatment planning CT due to scatter in cone beam geometry, slower acquisition, and/or the use of megavoltage radiation. However, the general features and geometry of the images are identical, allowing for direct comparison of the patient at treatment with the patient as he/she was planned. An additional potential advantage of volumetric CT scanning is the possibility of dose reconstruction or adaptive replanning.[6] Some targets such as prostate, cervix, and bladder can move significantly relative to the bony structures that are conventionally used for patient setup. Therefore, visualization of the tumor itself can reduce targeting uncertainty and therefore the required margin. Consequently, several protocols for clinical trial where high radiation doses are given require daily image guidance as a prerequisite for patient enrolment (http://clinicaltrialsfeeds.org/clinical-trials/show/NCT00304759). Not surprisingly, people are thinking how ‘target imaging’ in the treatment room can be made even more accurate than CT by implanting fiducial markers[6] or even using magnetic resonance imaging in the treatment room.[7]
3. Motion management finally adapts the treatment delivery to motion of the target during treatment.[8] The most important and most widely studied motion is breathing motion, and strategies for motion management include breath hold, gated delivery, and motion-adaptive radiotherapy.[9] It is important to note that motion management may not necessarily require intervention during treatment. By employing a method to examine motion during the treatment planning process, one can predict the locations where the target may be during the treatment. This can yield tighter margins than the guessing of motion due to ignorance. It is interesting to note that this information about an internal target volume (ITV)[2] is also provided by very slow imaging techniques that average overall positions of the moving target. Positron emission tomography is an example of this.[10]

It is now well established for many tumors that higher doses delivered to the target result in higher tumor control probability. Examples are prostate,[11] lung,[12] and liver[13] cancer.

However, dose escalation will in most cases be safe only if the margins can be reduced. Therefore, it is interesting, and perhaps not surprising, to note that several of the papers in the present issue of JMP are dedicated to at least one of the technologies listed above.

All these new approaches provide scope for reducing margins for radiotherapy treatment. However, careful consideration must be given to the distinction between CTV and PTV margins. Following the ICRU, the CTV consists of the gross tumor volume (GTV) with a margin to allow for subclinical extensions that ‘cannot be detected by the staging procedures.’[2] In practice, margins for GTV to CTV extension (clinical) and CTV to PTV extension (physical) have been added, with the result that the encompassing PTV will take care of both clinical and physical extensions. As technology provides us with the opportunity to reduce physical PTV margins, the irradiated volume becomes smaller; however, as we never had the possibility to determine the border between CTV and PTV margins from clinical data, we may reduce the coverage of subclinical disease.

Finally, it is important to note that critical structures may also be affected by setup uncertainty and movement. As such, the ICRU has introduced a concept of margins also for planning organ-at-risk volumes (PRVs).[2] As is the case for target structure, critical organs can also move and change their shape. Similar to PTV, internal and setup margins can also be defined.[2] For example, parotids may change shape and location, both of which are important to know at the time of aiming to deliver parotid-sparing radiotherapy for head and neck cancer. In practice, PRV margins are usually applied only for organs where sparing is critical, such as the spinal cord. However, PTVs and PRVs are not anatomical structures but are virtual structures that aid the radiotherapy treatment planning process. Therefore, they can overlap. This is one of the most challenging problems in treatment planning; and any method to reduce margins, even by a small amount, may significantly improve the treatment optimization options.

Only time will tell what impact margin reduction has on cancer treatment; there is no doubt that image guidance and IMRT reduce normal tissue complications and side effects of radiation. However, it will be essential to monitor progress and follow patients for a long term in prospective clinical trials to ascertain the true impact of margin reductions afforded by modern technology.
